# Advancing breeding phenology does not affect incubation schedules in chestnut‐crowned babblers: Opposing effects of temperature and wind

**DOI:** 10.1002/ece3.3524

**Published:** 2017-12-05

**Authors:** Elliot Capp, Andrea L. Liebl, Alexandra G. Cones, Andrew F. Russell

**Affiliations:** ^1^ UNSW Arid Zone Research Station School of Biological, Earth and Environmental Sciences University of New South Wales Sydney NSW Australia; ^2^ Centre for Ecology and Conservation College of Life and Environmental Sciences University of Exeter Penryn, Cornwall UK; ^3^ Department of Biology University of South Dakota Vermillion SD USA

**Keywords:** allometry, climate change, incubation bouts, incubation constancy, nest attentiveness, recess bouts

## Abstract

Projecting population responses to climate change requires an understanding of climatic impacts on key components of reproduction. Here, we investigate the associations among breeding phenology, climate and incubation schedules in the chestnut‐crowned babbler (*Pomatostomus ruficeps*), a 50 g passerine with female‐only, intermittent incubation that typically breeds from late winter (July) to early summer (November). During daylight hours, breeding females spent an average of 33 min on the nest incubating (hereafter on‐bouts) followed by 24‐min foraging (hereafter off‐bouts), leading to an average daytime nest attentiveness of 60%. Nest attentiveness was 25% shorter than expected from allometric calculations, largely because off‐bout durations were double the expected value for a species with 16 g clutches (4 eggs × 4 g/egg). On‐bout durations and daily attentiveness were both negatively related to ambient temperature, presumably because increasing temperatures allowed more time to be allocated to foraging with reduced detriment to egg cooling. By contrast, on‐bout durations were positively associated with wind speed, in this case because increasing wind speed exacerbated egg cooling during off‐bouts. Despite an average temperature change of 12°C across the breeding season, breeding phenology had no effect on incubation schedules. This surprising result arose because of a positive relationship between temperature and wind speed across the breeding season: Any benefit of increasing temperatures was canceled by apparently detrimental consequences of increasing wind speed on egg cooling. Our results indicate that a greater appreciation for the associations among climatic variables and their independent effects on reproductive investment are necessary to understand the effects of changing climates on breeding phenology.

## 1 INTRODUCTION

Changing patterns of climate over recent decades are having significant impacts on a range of organisms (Candolin & Wong, 2013). One of the most widely documented impacts is the emerging mismatch in the breeding phenology of predators and prey (Both, Van Asch, Bijlsma, Van Den Burg, & Visser, 2009; Thackeray et al., 2016; Visser, Both, & Lambrechts, 2004; Visser, van Noordwijk, Tinbergen, & Lessells, 1998). This mismatch is thought to arise primarily because prey, with their shorter generation times, are able to advance their reproductive cycle more rapidly in response to warming springs than predators (Both et al., 2009; Nussey, Postma, Gienapp, & Visser, 2005; Thackeray et al., 2016). Additionally, however, the strength of selection acting on advancing phenology might be expected to differ between ectothermic prey and endothermic predators. This is because shifting climates are also associated with increased climatic variability, leading early breeders to be increasingly exposed to periods of inclement weather (Vasseur et al., 2014). Oviparous endotherms, such as birds, might be particularly sensitive to inclement weather because of the necessity to maintain egg temperatures above critical thresholds during incubation (Webb, 1987; Visser ME, te Marvelde L & Lof ME, 2015). Although ambient temperature is known to have significant effects on incubation schedules in birds (Deeming, 2002), how lay‐date relates to key climatic variables experienced during incubation, and how these in turn impact incubation schedules are not well known (Hilde, Pélabon, Guéry, Gabrielsen, & Descamps, 2016; Marasco & Spencer, 2015).

In passerine birds, the female commonly performs all incubation, and so alone is responsible for raising egg temperature to the optimal 34–38°C required for embryonic development (Lea & Klandorf, 2002; Webb, 1987). Further, because her partner (or any other group members) seldom provisions enough to sustain the female while on the nest (Nord & Williams, 2015; Williams, 1996), the female must leave periodically to forage (Deeming, 2002; Skutch, 1957). This necessity leads to an obvious time allocation trade‐off between self‐maintenance and parental investment (Reid, Monaghan, & Nager, 2002). Although this trade‐off can be influenced by a number of factors, including food availability, partner provisioning, and nest structure (Conway & Martin, 2000; Marasco & Spencer, 2015; Martin & Ghalambor, 1999), climatic variables typically have the most profound effects (Carey, 2002; Deeming, 2002; Marasco & Spencer, 2015; Skutch, 1962). Low ambient temperature, in particular, is expected to pose a significant challenge for uni‐parental intermittent incubators (as exhibited by most passerines) because both female metabolic demands and egg cooling rates are increased (Carey, 2002; Hainsworth & Vos, 2002). Such effects might be further influenced by wind speed and relative humidity, but the effects of these on incubation behavior are less well documented (Deeming, 2002; Goldstein, 1983; Hilde et al., 2016; Marasco & Spencer, 2015).

The metabolic costs of incubation are well established, particularly in uni‐parental incubators that need to reheat their eggs following off‐bouts. For example, during incubation, field metabolic rates are typically 2–3 times higher than basal levels, and in small uni‐parental incubators, energetic expenditure during incubation is comparable to that during nestling provisioning (Nord & Williams, 2015; Tatner & Bryant, 1993; Tinbergen & Williams, 2002; Williams, 1996). In addition, supplemental feeding experiments during incubation are associated with increased nest attentiveness and reduced foraging durations (Nilsson & Smith, 1988), and experimental cooling during incubation can lead to reduced adult body condition and immune function (Ardia, Pérez, & Clotfelter, 2010). Although females can mitigate these costs by building more insulated nests early in the season (Hatchwell, Fowlie, Ross, & Russell, 1999), an obvious alternative way to reduce incubation costs is to breed later in the year when climatic conditions are more suitable. As such, incubation costs, particularly for passerines that show female‐only intermittent incubation, might act as a significant check on the evolution of advancing breeding phenology in response to warming springs (Visser et al. 2015).

**Figure 1:**
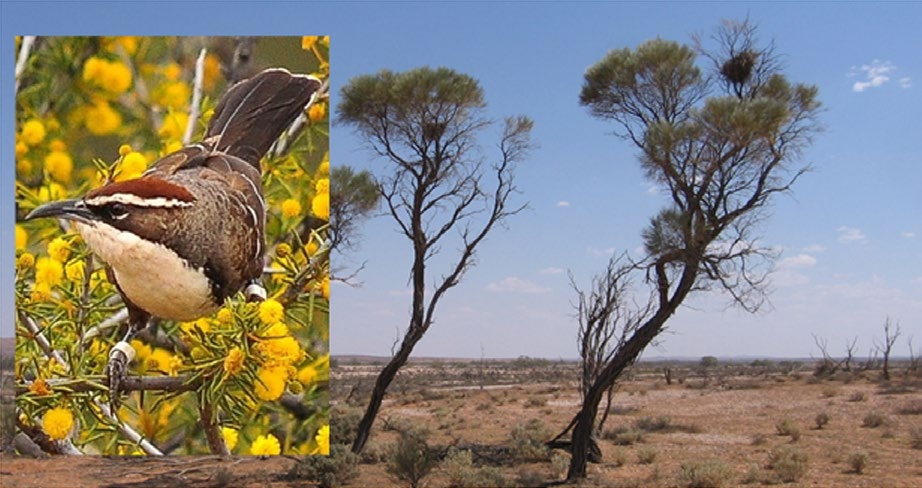
Chestnut‐crowned babbler, a 50 g endemic passerine of inland southeastern Australia inset in its natural arid habitat at Fowlers Gap, with two babbler nests in classic position. Like most passerines, a single female is responsible for all incubation at each nest and must balance the need to incubate eggs with the need to forage [photograph credits Niall Stopford (babbler) & Ian Stewart (landscape)]

To examine the validity of this hypothesis, we investigate the relationships among breeding phenology, climate and incubation schedules in the chestnut‐crowned babbler (*Pomatostomus ruficeps*), a 50 g insectivorous passerine (Figure 1) endemic to inland regions of southeastern Australia. Like most passerines, this species shows intermittent female‐only contact incubation (Young, Browning, Savage, Griffith, & Russell, 2013). Breeding occurs in domed nests made of twigs and lined with mud, herbivore feces, soft vegetation, and some wool or feathers (Chappell, Buttemer, & Russell, 2016). On average, females lay clutches of four eggs, each egg weighing ca. 4 g, which hatch following ca. 20 days of incubation (Russell, Portelli, Russell, & Barclay, 2010). Egg laying typically begins in late winter (July) and ends in early summer (November), with peaks in August and September (Russell, 2016). Early breeding is likely to be energetically expensive, as resting metabolic rates for lone individuals in nests at 5°C and 15°C (typical of early and median lay dates) are 86% and 40% greater, respectively, than they are at thermal neutral temperatures (i.e., 28–32°C) (Chappell et al., 2016). On the other hand, there are two key benefits from breeding early in the season. First, the primary food of nestling babblers (i.e., lepidoptera and coleopteran larvae (Browning et al., 2012)) emerges in response to winter rain. Second, early breeding allows multiple breeding attempts before the prohibitively hot summer months (Russell et al., 2010).

We have four specific aims. First, we describe the relationships between breeding phenology and average climatic conditions experienced during the 20‐day incubation period. Second, we investigate the effects of climate for both on‐ and off‐bout durations, as well as any mediating effects of clutch size and embryo age. Third, using temperature gauges in model eggs at a subset of nests, we tested the effects of ambient climate on egg cooling rates. Finally, we determine the relationship between breeding phenology and overall nest attentiveness behavior, and determine the underlying climatic causes of the variation observed.

## 2 MATERIALS AND METHODS

### 2.1 Study site and climate

The study was carried out in the breeding season of 2013 (July–November) at the University of New South Wales Arid Zone Research Station (Fowlers Gap) in western New South Wales, Australia (31°05′S, 141°43′E). The habitat is dominated by open chenopod shrubland, with short linear stands of trees largely confined to drainage lines and creek beds that descend from the surrounding Barrier Ranges (see Portelli, Barclay, Russell, Griffith, & Russell, 2009; Russell et al., 2010 for full details). Temperature, wind speed, and relative humidity were collected on‐site on the hour by an Australian Bureau of Meteorology weather station. Rainfall was practically nonexistent during the study, never exceeding 5 mm over 24 hr and was not considered here.

From 01 July to 22 November, encompassing periods of incubation observed in 2013, the average temperature each hour (6 a.m.–6 p.m.) was 19.9°C (hourly range = −0.8 to +40.4, *SD* = ±7.7°C, *n* = 1,849 hourly measures); the average hourly wind speed was 18.6 km/hr (hourly range = 0–50, *SD* = ±10 km/hr); and the average hourly relative humidity was 36% (hourly range 4%–99%, *SD* = ±21%). Low temperatures of <10°C occurred in 10% of hours, with 50% of these in July and 33% in August, whereas thermal neutral temperatures in excess of 27°C were absent in July, rare in August (<5%), and increasingly common in the subsequent months. In turn, relatively high winds in excess of 20 km/hr occurred over 44% of hours, whereas wind speeds under 10 km/hr occurred during 20% of hours. Wind speeds below 10 km/hr were most common in July and August (63%), while those in excess of 20 km/hr were least common in July (16%) and most common in October (29%), with comparable prevalence in other months. Thus, hourly temperatures and wind speeds were positively correlated over the daylight periods considered (*r*_p_ = 0.42, *n* = 1,849 hourly measures of each, *p* < .001), while relative humidity levels declined throughout the season in association with increasing temperatures (*r*_p_ = −0.68, *p* < .001).

### 2.2 Nest observations

Overall, we performed 50 nest observations (totaling 315 hr) at 16 breeding nests of 14 breeding units, with each nest being observed on 2–4 occasions. All such observations were conducted from 08 August to 06 October and encompassed most hours of daylight (i.e., first light to late afternoon). Observations were either conducted by a single observer (E.C.) positioned in a concealed position with a scope 30–50 m from the nest, usually in conjunction with a video camera (Sony HDR‐CX220) (*N* = 25 observations, 0.75–4.2 hr in duration, mean = 3 hr) or using a video camera alone (*N* = 25 observations, duration range = 6–12.4 hr, mean = 9.6 hr). There was no difference in nest attentiveness using the two methods (*t* test: *t*_48_ = 0.72, *p* = .48). During each observation date, we recorded time of day, and female entrance and exit times from the nest.

Observation periods necessarily started part‐way through either an on‐ or an off‐bout, but always ended with either a nest exit or entrance; in the analyses, we used only complete bouts. Consequently, on‐bouts were determined as the time between female entrance and exit from the nest, while off‐bouts were defined as the time between exit and subsequent entrance. The one caveat was that bouts of either were only defined when they exceeded 2 min in duration (see below). Overall, we obtained 284 complete on‐bouts and 283 complete off‐bouts, across the 50 observation periods of the 16 breeding attempts. Finally, we also translated the time on versus off the nest during each observation period into an index of daytime nest attentiveness using Kendeigh's (1952) equation:

$$\frac{\mathrm{mean}\;\mathrm{on}-\;\mathrm{bout}\;\mathrm{durations}}{(\mathrm{mean}\;\mathrm{on}-\;\mathrm{bout}\;\mathrm{durations}+\mathrm{mean}\;\mathrm{off}-\;\mathrm{bout}\;\mathrm{durations})}\times 100.$$

This generated a single value of nest attentiveness for each nest on each day of incubation.

### 2.3 Verification of active incubation and climatic effects on egg cooling during off‐bouts

To verify that nest entrance and exits are indicative of the duration of active incubation in chestnut‐crowned babblers, we inserted a model egg containing a small temperature gauge (Sigma Delta Technologies) into five nests. Model eggs were made from plaster of Paris strips wrapped around individual gauges; we routinely use model eggs in babbler nests without ill‐effect (E. C. Berg & A. F. Russell unpubl. results) and whether or not nests contained model eggs failed to influence incubation schedules in any analysis in this study (results not shown). The gauges measured temperature every 2 min to within 0.01°C. Using this method, we obtained precise temperature measures from within the nest for 8–20 days of incubation at the five nests. Matching egg temperature data to observed on‐bouts confirmed that active incubation occurred during all on‐bouts, although occasional on‐ and off‐bouts of 2 min (or less) typically failed to generate reliable changes in egg temperatures. Thus, occasional on‐ and off‐bouts of under 2 min were excluded from calculations of on‐ and off‐bout durations (see above).

We also used these gauges to examine the effects of climate on egg cooling rates during off‐bouts. Although the plaster eggs do not provide quantitative assessment of precise cooling rates of real eggs, they are suitable to assess the qualitative, and relative, impacts of the key climatic variables (i.e., temperature, wind speed, and relative humidity). Off‐bouts were identified as consistent drops in model egg temperature using a combination of Rhythm (Cooper & Mills, 2005) and Raven (Cornell Lab of Ornithology 2013) software, with the latter showing a waveform of the temperature variations. Our assignment of off‐bouts using this method was confirmed for the subset where we also had direct observations of female incubation behavior (see above). From this model egg temperature data, we obtained the time at each off‐bout onset and matched these times to the ambient climate data (to the nearest hour). Climatic effects on egg cooling rates were investigated during 696 identified off‐bout periods over 74 nest days at the five nests [mean = 9.4 recess periods per day ±5.6 (*SD*)].

### 2.4 Statistical analyses

All analyses were conducted in Genstat v. 17 (VSN International). First, we investigated the effects of lay‐date on the mean daily temperature and wind speed over the course of their 20‐day incubation period using separate Spearman's rank correlations. For these purposes, we consider incubation onset dates between 01 July and 02 November 2013, and the mean daily climatic conditions experienced between 6 a.m. and 6 p.m. over the subsequent 20 days (i.e., encompassing the 20‐day incubation period at each nest). Second, we used residual maximum likelihood (REML) models to investigate the factors affecting the duration of both on‐ and off‐bouts. In each case, the term of interest was fitted as the response term to a normal error structure following square root (on‐bouts) and natural log (off‐bouts) transformations. The explanatory terms included were climatic variables (temperature, wind speed, and relative humidity to the nearest hour during the onset of each on‐ and off‐bout), clutch size (range = 3–5, mode = 4), embryo age (range = 1–20 days from incubation onset, mean = 11 days), and the duration of the previous off‐bout (both analyses) or on‐bout (off‐bout analysis). Observation day and nest identities were fitted as random intercepts to adjust for repeated measures of each. Time of day was not included as both temperature and wind speed increase during the day, and preliminary analyses suggested that it had no impact on either on‐ or off‐bout durations. Third, we used REML to investigate climatic effects on egg cooling rates, with the drop in egg temperature during each off‐bout fitted as the response term following square root transformation. In this case, we fitted off‐bout duration (log transformed), the difference in temperature between egg and ambient at off‐bout onset, wind speed and humidity as explanatory terms, and nest identity as a random intercept. In this analysis, all climatic terms were *z*‐transformed to offer direct comparison of the magnitude of each effect on egg cooling (Schilzeth, 2010). Finally, we also used a REML to investigate the influence of lay‐date on nest attentiveness during the hours of daylight. Here, nest attentiveness was fitted as the response term and observation duration, lay‐date, clutch size, and embryo age were fitted as explanatory terms, while nest identity was fitted as a random intercept. Explanatory terms were removed from all models if they failed to increase explanatory power based on log‐likelihood estimates (Zuur, Leno, Walker, Saveliev, & Smith, 2009).

## 3 RESULTS

### 3.1 Breeding phenology and climate during incubation

**Figure 2:**
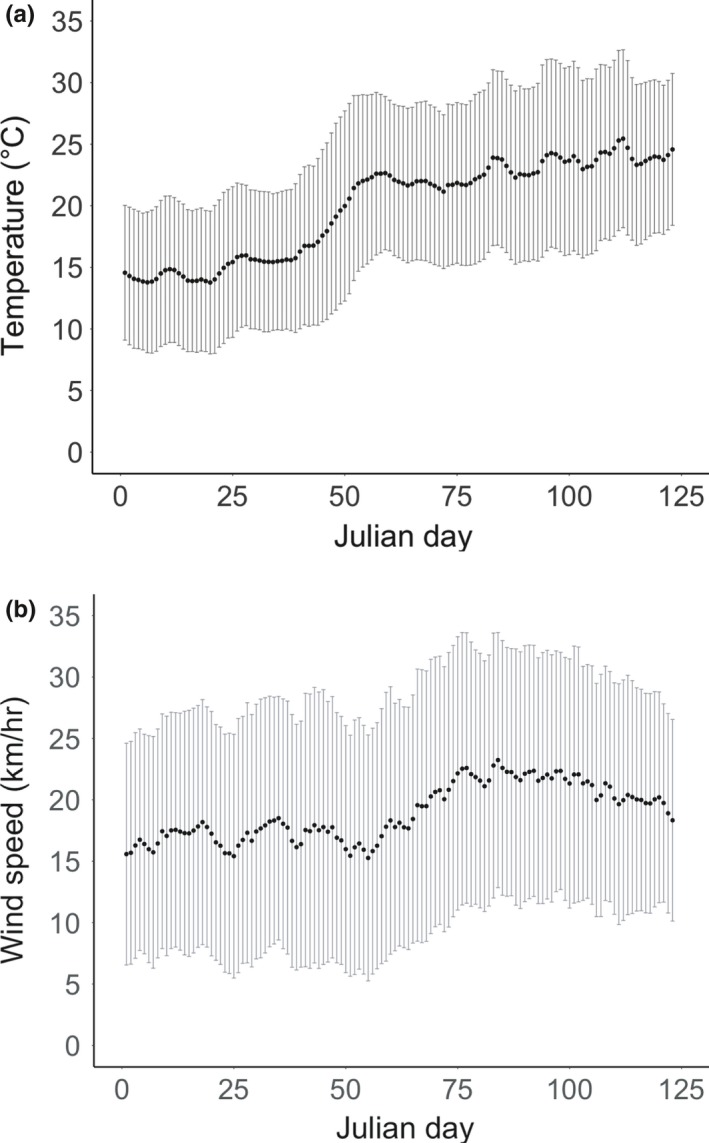
Association between the date of incubation onset and the average (±*SD*) daily temperature (a) and wind speed (b) experienced over the subsequent 20 days of incubation. Delaying breeding phenology was associated with both increasing temperatures and wind speeds experienced during incubation (temperature: *r*_s_ = 0.95, *p* < .001; wind: *r*_s_ = 0.74, *p* < .001)

Advancing lay‐date toward 01 July from 02 November was associated with significant reductions in both temperature and wind speed experienced during the 20‐day incubation period (Figure 2). Overall, for every day that incubation onset was advanced, the mean average daily temperature experienced over the 20‐day incubation period decreased by ca. 0.1°C, generating an average maximum daily temperature difference of 12°C experienced by the earliest and latest layers during incubation (Figure 2a). There was also evidence for a step change in incubation temperatures experienced: Incubation periods initiated before 13 August versus after 20 August had average daytime temperatures of 12–16°C versus 20–25°C, respectively. Over the same 4‐month period, average wind speed during incubation increased by an average of 0.05 km/hr per day, from an average low of 15 km/hr during early incubation periods to an average high of 23 km/hr for those laying from September (Figure 2b). Again, there was some suggestion of a step increase in wind speed, with average speeds of 15–17 km/hr during incubation in July/August compared with 20–23 km/hr during incubation in September/October. Thus, early laying was associated with low temperatures and low wind speeds during incubation, with increases in each being experienced by later breeders; particularly for those laying from around mid‐August.

### 3.2 Factors affecting on‐ and off‐bout durations

**Figure 3:**
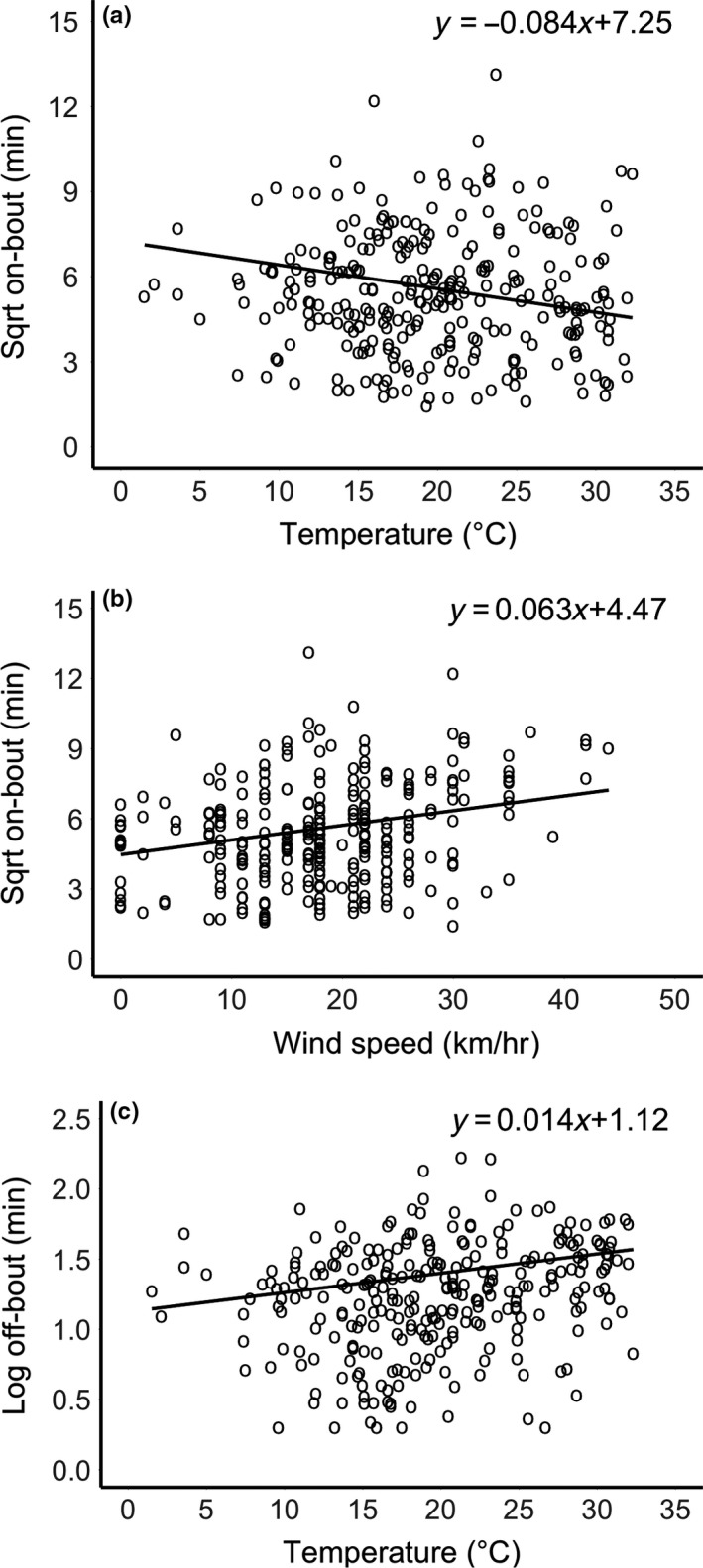
Climatic effects on on‐bout and off‐bout durations. On‐bout durations (a) decreased with increasing temperature and (b) increased with increasing wind speed. (c) Off‐bout durations increased with increasing temperature. Figures show raw data with predicted regression line; see Tables 1 and 2 for statistics

On‐bout durations varied from 2 min (as defined) to 171 min, with an average duration of 33 min (*SD* = ±26 min). This variation was significantly affected by the duration of the previous off‐bout, current temperature, and current wind speed, but there were no effects of relative humidity, clutch size, or embryo age (Table 1). On‐bout durations increased 3.5‐fold across the range of previous off‐bout durations, from an average low of 20 min following a 2 min recess to an average high of 90 min following exceptional recess periods of close to 3 hr. The effects of temperature and wind speed were only slightly more modest. At lows of 2°C, incubation bouts averaged ca. 50 min, but at 32°C they were 50% shorter, averaging ca. 25 min (Figure 3a). Similarly, in the absence of wind, incubation bout durations averaged 20 min, but in winds of 45 km/hr, these durations increased 2.5‐fold to average ca. 50 min (Figure 3b).

**Table 1:** Factors affecting the duration of on‐bout durations. Estimates provided following square root transformation. Random term (nest identity: coefficient = 0.55 ± 0.29 *SE*)

	Estimate ± *SE*	*F* Statistic	*df*	*p* value
Previous recess duration	0.030 ± 0.0056	26.45	266	<.001
Current temperature	−1.12 ± 0.35	9.77	232	.002
Current wind speed	0.061 ± 0.014	20.61	267	<.001
Current humidity	−0.0036 ± 0.012	0.09	246	.76
Clutch size	0.42 ± 0.40	1.07	11	.32
Days from hatching	−0.018 ± 0.034	0.26	89	.61
Constant	5.48 ± 0.22			

Off‐bout durations varied from 2 min (as defined) to 167 min, with an average of 24 min (*SD* = ±22 min). Variation in off‐bout duration was explained by the duration of the previous on‐bout and by current temperature, but there were no effects of the duration of the previous off‐bout, wind speed, relative humidity, clutch size, or embryo age (Table 2). Minimum on‐bout durations were associated with subsequent off‐bouts averaging 15 min, as opposed to extreme on‐bouts of close to 3 hr, which were associated with twofold increases in subsequent off‐bout durations (ca. 32 min). After controlling for this effect, we found that off‐bouts averaged just 12 min at low temperatures (i.e., 2°C) but increased threefold to 40 min at 32°C (Figure 3c).

**Table 2:** Factors affecting the duration of off‐bouts. Estimates provided following natural logarithm transformation. Random term (nest identity: coefficient = 0.016 ± 0.009 *SE*)

	Estimate ± *SE*	*F* Statistic	*df*	*p* value
Previous on duration	0.0026 ± 0.00086	8.34	278	.004
Current temperature	0.014 ± 0.0039	12.48	185	<.001
Current wind speed	0.0022 ± 0.0027	0.67	279	.41
Current humidity	0.00069 ± 0.0024	0.08	222	.78
Clutch size	0.095 ± 0.066	0.095	10	.18
Days from hatching	0.0021 ± 0.0060	0.13	99	.72
Previous recess duration	−0.00012 ± 0.0011	0.01	265	.92
Constant	1.24 ± 0.040			

### 3.3 Climatic effects on egg cooling rates

Off‐bout durations using the model egg method aligned well with the observational method used above, with average off‐bouts of 27 ± 27 (*SD*) mins (compared to 24 ± 22 min using the observational method). During off‐bouts, model eggs dropped in temperature by an average of 5.3 ± 3.3°C (*SD*) (Table 3). Unsurprisingly, the duration of the off‐bout (log transformed) had a substantial effect on the magnitude of egg cooling. After controlling for this effect, we found a significant detrimental effect of the difference in temperature between the model egg and ambient at off‐bout onset: There was an additional 2°C drop in model egg temperature for every extra 7°C difference between initial egg temperature and ambient temperature over the average 27‐min off‐bout duration. Further, increasing wind speed also significantly impacted egg cooling particularly at low temperatures: On average, a 10 km/hr increase in wind speed was associated with an extra drop in temperature of 0.3°C per min; the magnitude of this effect increased by a further 0.4°C per min for every 7°C increase in temperature differentials between egg and ambient. Finally, there was also a marginal benefit of high relative humidity on reducing egg cooling (Table 3). Although we cannot equate these effects to real eggs, it is likely that cooling rates in nature are primarily impacted by long recess periods and low ambient temperatures and that high wind speed particularly at low temperatures has a further influence.

**Table 3:** Factors affecting cooling of model eggs during off‐bouts. Temperature difference (temp diff) reflects the difference between egg and ambient temperature at off‐bout onset. All explanatory terms were *z*‐transformed, and estimates are provided following square root transformation. Estimates and statistics for temperature difference and wind speed are provided without the interaction. Random term (nest identity: coefficient = 0.25 ± 0.20 *SE*)

	Estimate ± *SE*	*F* Statistic	*df*	*p* value
Recess duration	2.098 ± 0.075	782.43	715	<.001
Temperature difference	2.018 ± 0.11	318.73	716	<.001
Current wind speed	0.28 ± 0.083	11.23	715	<.001
Current humidity	−0.22 ± 0.11	3.99	713	.046
Temp diff × wind speed	0.40 ± 0.065	36.67	713	<.001
Constant	5.39 ± 0.24			

### 3.4 Breeding phenology and nest attentiveness

**Figure 4:**
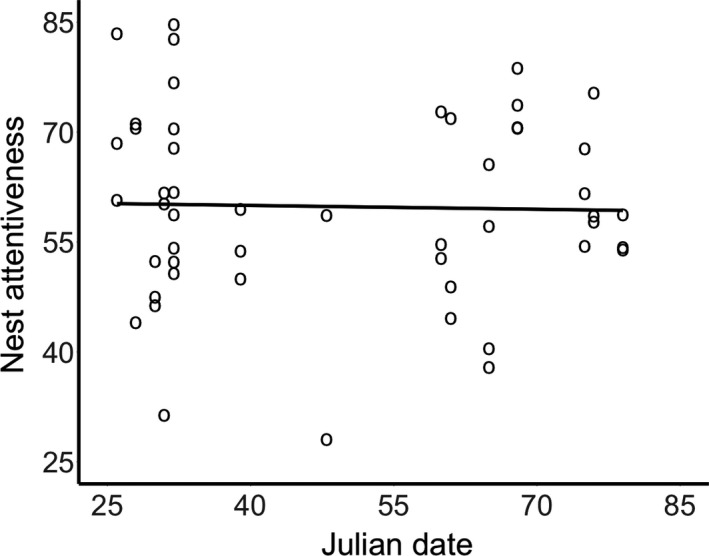
Breeding phenology and nest attentiveness. We found no evidence to suggest that daytime nest attentiveness was influenced by the date of incubation onset (residual maximum likelihood: *F*_1,29_ = 0.38, *p* = .54). Figure shows raw data and predicted regression line (*y* = −0.017*x* + 60.7)

The percent of observation hours females were found incubating varied from 28% to 85% among observation days (mean = 60% ± 13% *SD*) and from 43% to 83% among nests (mean = 60% ± 10% *SD*). However, we found little evidence to suggest that nest attentiveness on a given day was influenced by lay‐date (Figure 4). To investigate this lack of relationship further, we replaced lay‐date with average daily temperature and wind speed experienced during each observation day. While increasing temperatures were associated with reduced nest attentiveness (*F*_1,44_ = 4.01, *p* = .051; estimate ±*SE* = −0.58 ± 0.29), increasing wind speeds were associated with increased nest attentiveness (*F*_1,47_ = 14.5, *p* < .001; estimate ±*SE* = 0.78 ± 0.21). It thus appears that any benefit of increasing temperatures with progression of the season is canceled by the costs of increasing wind speed. We found no additional effects of observation duration (*F*_1,37_ = 0.04, *p* = .84), relative humidity (*F*_1,44_ = 0.02, *p* = .88), clutch size (*F*_1,13_ = 0.00, *p* = .99), or embryo age (*F*_1,35_ = 0.00, *p* = .99) on nest attentiveness.

## 4 DISCUSSION

Chestnut‐crowned babbler breeding phenology had a profound effect on the climatic conditions experienced during incubation, with average daytime temperatures doubling and wind speed increasing by 50% from the beginning to end of the season. In turn, both temperature and wind speed had significant effects on incubation schedules. For example, from the lowest to highest temperatures experienced, females halved their on‐bout durations and doubled their off‐bout periods. Similarly, from the lowest to highest wind speeds encountered, females doubled their on‐bout duration, although in this case off‐bout durations were unaffected. By contrast, we found no effects on either on‐ or off‐bout durations of relative humidity, clutch size, or embryo age. Finally, despite the substantial temperature gradient over the breeding period, and female responsiveness to variation in daytime weather patterns, we found no evidence to suggest that lay‐date impacted overall nest attentiveness. This puzzling result appears to be driven by the positive association between temperature and wind speed and the opposing effects of each on on‐bout durations.

The average nest attentiveness level found in this study is within the range shown in other female‐only incubators (45%–100%). However, at 60%, it is below the average of 73% (±11% *SD*) of passerine incubators, as well as the predicted 73%–74% based on egg mass (4 g each in babblers) and clutch mass of 16 g (Deeming, 2002). There are at least three potential reasons for the relatively low nest attentiveness levels shown in babblers. First, the low relative humidity levels typical of arid environments (mean = 36% during this study) might select for reduced egg shell porosity to reduce transpiration (Ar & Rahn, 1985). If this is the case in babblers, developmental rates might be more constrained by the transfer of oxygen rather than heat, leading to a reduced need for females to have high attentiveness. Second, the relatively hot conditions experienced by developing embryos during our observations up to 32°C, coupled with the favorable microclimate provided by dome‐shaped nests (Chappell et al., 2016; Mainwaring, 2015), could have resulted in a reduced need for greater attentiveness (Serventy, 1971). Indeed, Griffith, Mainwaring, Sorato, and Beckmann (2016) showed that zebra finch (*Taeniopygia guttata*) eggs laid at the same site, and which also develop in domed nests, could be hatched successfully without the need for any incubation, albeit after a protracted period. Third, low food availability typical of arid zones might require females to take protracted off‐bouts to sustain their own metabolic demands, constraining them from being more attentive (Serventy, 1971).

Although we have yet to quantify egg shell porosity in babblers, we can shed some light on whether low nest attentiveness stems from a temperature‐mediated relaxation of the need to be more attentive (here‐named on‐bout relaxation hypothesis) or a constraint imposed by the need for long off‐bouts (here‐named foraging constraints hypothesis). The key predictions of the on‐bout relaxation hypothesis are that on‐bout durations should be less than expected (for a bird laying a 4 g egg, 16 g clutch), but that the duration of the entire incubation period should be as predicted for a species with this egg and clutch mass (as reduced on‐bout durations at high temperatures should not impact overall incubation duration). We find partial support: On‐bout durations were 25% shorter than expected (33 min vs. the 45–47 min predicted, Deeming, 2002), but the duration of the incubation period, at 20 days, is 18% longer than expected (Ricklefs & Starck, 1998). Together these results suggest that ambient temperatures coupled with any beneficial effects of breeding in a domed nest are not sufficiently high, on average, to account for the reduced nest attentiveness. It therefore seems likely that the time taken to recoup sufficient energy during off‐bouts provides an additional explanation. In support of the foraging constraints hypothesis, off‐bouts were double (mean = 24 min) those expected based on egg and clutch mass (expected = 11–12 min) (Deeming, 2002). Thus, it appears that the need for long off‐bouts constrains nest attentiveness in chestnut‐crowned babblers and that females use warm times of the day to increase off‐bout durations to mitigate the costs (Biebach, 1986; Boulton et al. 2010; Tulp & Schekkerman, 2006; Vleck, 1981).

That birds are responsive to ambient temperature when making decisions to remain on or off the nest is a common, although not universal, finding in species with intermittent, contact incubation (Conway & Martin, 2000; Deeming, 2002; Skutch, 1962). What is more surprising in this study is that incubation behavior in female chestnut‐crowned babblers is also highly responsive to wind speed. The effects of wind speed on incubation behavior are seldom considered (Cartar & Montgomerie, 1987; Hilde et al., 2016; Tulp & Schekkerman, 2006), but we show here that its effects on on‐bout durations and nest attentiveness are comparable to the effects of temperature. The impacts of wind speed on on‐bout durations and attentiveness might manifest through reduced foraging efficiency or increased egg cooling (Goldstein, 1983). If the effect of increasing wind speed was to manifest through compromised foraging efficiency, then we would expect to find a positive association between wind speed and off‐bout duration—this was not the case. Instead, wind speed was positively associated with on‐bout duration, suggesting that wind had an exacerbating effect on egg cooling rates. In support, cooling of model eggs during recess periods was accelerated by increasing wind speed, particularly at low temperatures. Conway and Martin (2000) suggested that the impact of climatic parameters on incubation behavior should manifest more in off‐bout (than on‐bout) durations due to their influence on egg cooling rates, but here we show that when it comes to wind speed it is only the on‐bout duration that is affected. Further, our wind speed effects are suitably strong to influence temperature effects, leading to the suggestion that wind speed is not only an important parameter in studies of incubation behavior but might help to explain the lack of temperature effects on nest attentiveness in some species (Deeming, 2002).

In our study, ambient temperature and wind speed positively covaried over the breeding season. This covariation, and the antagonistic effect each had on incubation behavior, explains the lack of relationship between breeding phenology and nest attentiveness. In this system, therefore, our current evidence suggests that, all else being equal, breeding phenology and incubation investment are unlinked: The incubation costs of early breeding imposed by low temperatures are mitigated by low wind speeds, whereas the benefits of late breeding arising from higher temperatures are offset by higher wind speeds. Although we have shown previously that the metabolic costs of individually roosting (as a breeding female would do) at 5°C are substantially higher than at 15°C, which in turn are substantially greater than at thermal neutral temperatures (28–32°C) (Chappell et al., 2016), we were unable to test the exacerbating effects of wind speed on metabolic costs. We propose further metabolic studies are required to fully elucidate the relationship between breeding phenology and the costs of incubation, as well as the mediating effects of temperature and wind, to understand selective impediments to advancing lay‐dates under a changing climate. Notwithstanding, our take‐home point is that understanding the relationships among climatic variables, and the impacts that each has on incubation, are required to make coherent predictions regarding the effects of breeding phenology on the costs of incubation.

In general, breeding phenology is likely to associate with a number of defining climatic parameters, including temperature, wind speed, and rainfall, as well as nest structure (Deeming, 2002; Mainwaring, 2015; Marasco & Spencer, 2015). In northern temperate latitudes, wherein most of the research on incubation behavior in intermittent contact species has been conducted, temperature and wind speed (and rainfall) might be more likely to covary negatively (rather than positively) over the breeding season. Our results suggest that such negative covariation will have substantial effects on the costs of incubation early in the breeding season. This is not only because low temperatures coupled with high wind speeds will have synergistic effects on egg cooling rates, but because off‐bouts will need to be shorter as a consequence. In combination, such effects are likely to act as a significant impediment to selection on early reproduction in passerine birds with female‐only, intermittent incubation. While the current mismatch in breeding phenology of northern passerines and their insect prey has been chiefly explained by the formers’ longer generation time, our results suggest that an additional factor is the costs of incubation for early laying females.

## CONFLICT OF INTEREST

None Declared.

## AUTHOR CONTRIBUTIONS

The study was conceived and performed by EC, ALL, and AFR; analyzed by AFR and AGC; and written by EC, ALL, and AFR.

## References

[ece33524-bib-0001] Ar, A. , & Rahn, H. (1985). Pores in avian egg shells: Gas conductance, gas exchange and embryonic growth rate. Respiration Physiology, 61, 1–20. 4035113 10.1016/0034-5687(85)90024-6

[ece33524-bib-0002] Ardia, D. R. , Pérez, J. H. , & Clotfelter, E. D. (2010). Experimental cooling during incubation leads to reduced innate immunity and body condition in nestling tree swallows. Proceedings of the Royal Society of London. Series B: Biological Sciences, 277, 1881–1888. 20147326 10.1098/rspb.2009.2138 PMC2871872

[ece33524-bib-0003] Biebach, H. (1986). Energetics of rewarming a clutch in starlings (*Sturnus vulgaris*). Physiological Zoology, 59, 69–75.

[ece33524-bib-0004] Bioacoustics Research Program (2013). Raven Pro: Interactive Sound Analysis Software (Version 1.5) [Computer software]. Ithaca, NY: The Cornell Lab of Ornithology. Retrieved from http://www.birds.cornell.edu/raven.

[ece33524-bib-0005] Both, C. , Van Asch, M. , Bijlsma, R. G. , Van Den Burg, A. B. , & Visser, M. E. (2009). Climate change and unequal phenological changes across four trophic levels: Constraints or adaptations? Journal of Animal Ecology, 78(1), 73–83. 18771506 10.1111/j.1365-2656.2008.01458.x

[ece33524-bib-0500] Boulton RL, Richard Y , Armstrong DP . The effect of male incubation feeding, food and temperature on the incubation behaviour of New Zealand robins. Ethology. 2010;116:490–497.

[ece33524-bib-0006] Browning, L. E. , Young, C. M. , Savage, J. L. , Russell, D. J. F. , Barclay, H. , Griffith, S. C. , & Russell, A. F. (2012). Carer provisioning rules in an obligate cooperative breeder: Prey type, size and delivery rate. Behavioral Ecology and Sociobiology, 66(12), 1639–1649.

[ece33524-bib-0007] Candolin, U. , & Wong, B. B. M. (2013). Behavioural responses to a changing climate: Mechanisms and consequences. Oxford, UK: Oxford University Press.

[ece33524-bib-0008] Carey, C. (2002). Incubation in extreme environments In Deeming D. C. (Ed.), Avian incubation: Behaviour, environment and evolution (pp. 238–253). Oxford, UK: Oxford University Press.

[ece33524-bib-0009] Cartar, R. V. , & Montgomerie, R. D. (1987). Day‐to‐day variation in nest attentiveness of white‐rumped sandpipers. The Condor, 99, 252–260.

[ece33524-bib-0010] Chappell, M. A. , Buttemer, W. A. , & Russell, A. F. (2016). Energetics of communal roosting in chestnut‐crowned babblers: Implications for group dynamics and breeding phenology. Journal of Experimental Biology, 219, 3321–3328. 27807215 10.1242/jeb.144972

[ece33524-bib-0011] Conway, C. J. , & Martin, T. E. (2000). Evolution of passerine incubation behavior: Influence of food, temperature, and nest predation. Evolution, 54, 670–685. 10937242 10.1111/j.0014-3820.2000.tb00068.x

[ece33524-bib-0012] Cooper, C. B. , & Mills, H. (2005). New software for quantifying incubation behaviour from time‐series recordings. Journal of Field Ornithology, 76(4), 352–356.

[ece33524-bib-0013] Deeming, D. C. (2002). Behavioural patterns during incubation In Deeming D. C. (Ed.), Avian incubation: Behaviour, environment and evolution (pp. 63–87). Oxford, UK: Oxford University Press.

[ece33524-bib-0014] Goldstein, D. L. (1983). Effect of wind on avian metabolic rate with particular reference to Gambel's Quail. Physiological Zoology, 56(4), 485–492.

[ece33524-bib-0015] Griffith, S. C. , Mainwaring, M. C. , Sorato, E. , & Beckmann, C. (2016). High atmospheric temperatures and ‘ambient incubation’ drive embryonic development and lead to earlier hatching in a passerine bird. Royal Society Open Science, 3, 150371, 1–14. 10.1098/rsos.150371 PMC4785966 26998315

[ece33524-bib-0016] Hainsworth, F. R. , & Vos, M. A. (2002). Intermittant incubation: Predictions and tests for time and heat allocations In Deeming D. C. (Ed.), Avian incubation: Behaviour, environment and evolution (pp. 223–237). Oxford, UK: Oxford University Press.

[ece33524-bib-0017] Hatchwell, B. J. , Fowlie, M. K. , Ross, D. J. , & Russell, A. F. (1999). Incubation behavior of long‐tailed tits: Why do males provision incubating females? The Condor, 101, 681–686.

[ece33524-bib-0018] Hilde, C. H. , Pélabon, C. , Guéry, L. , Gabrielsen, G. W. , & Descamps, S. (2016). Mind the wind: Microclimate effects on incubation effort of an arctic seabird. Ecology and Evolution, 6, 1914–1921. 27099703 10.1002/ece3.1988 PMC4831427

[ece33524-bib-0019] Kendeigh, S. C. (1952). Parental care and its evolution in birds. Champaign, IL: University of Illinois Press.

[ece33524-bib-0020] Lea, R. W. , & Klandorf, H. (2002). The brood patch In Deeming D. C. (Ed.), Avian incubation: Behaviour environment and evolution (pp. 100–118). Oxford, UK: Oxford University Press.

[ece33524-bib-0021] Mainwaring, M. C. (2015). Nest construction and incubation in a changing climate In Deeming D. C., & Reynolds S. J. (Eds.), Nests, eggs and incubation: New ideas about avian reproduction (pp. 65–74). Oxford, UK: Oxford University Press.

[ece33524-bib-0022] Marasco, V. , & Spencer, K. A. (2015). Improvements in our understanding of behaviour during incubation In Deeming D. C., & Reynolds S. J. (Eds.), Nests, eggs and incubation: New ideas about avian reproduction (pp. 142–151). Oxford, UK: Oxford University Press.

[ece33524-bib-0023] Martin, T. E. , & Ghalambor, C. K. (1999). Males feeding females during incubation I: Required by microclimate or constrained by nest predation? The American Naturalist, 153, 131–139. 10.1086/303153 29578762

[ece33524-bib-0024] Nilsson, J.‐Å. , & Smith, H. G. (1988). Incubation feeding as a male tactic for early hatching. Animal Behaviour, 36, 641–647.

[ece33524-bib-0025] Nord, A. , & Williams, J. B. (2015). The energetic costs of incubation In Deeming D. C., & Reynolds S. J. (Eds.), Nests, eggs and incubation: New ideas about avian reproduction (pp. 152–170). Oxford, UK: Oxford University Press.

[ece33524-bib-0026] Nussey, D. H. , Postma, E. , Gienapp, P. , & Visser, M. E. (2005). Selection on heritable phenotypic plasticity in a wild bird population. Science, 310(5746), 304–306. 16224020 10.1126/science.1117004

[ece33524-bib-0027] Portelli, D. J. , Barclay, H. , Russell, D. J. F. , Griffith, S. C. , & Russell, A. F. (2009). Social organisation and foraging ecology of the cooperatively breeding chestnut‐crowned babbler (*Pomatostomus ruficeps*). Emu, 109, 153–162.

[ece33524-bib-0028] Reid, J. M. , Monaghan, P. , & Nager, R. G. (2002). Incubation and the costs of reproduction In Deeming D. C. (Ed.), Avian incubation: Behaviour, environment and evolution (pp. 314–325). Oxford, UK: Oxford University Press.

[ece33524-bib-0029] Ricklefs, R. E. , & Starck, J. M. (1998). Emryonic growth and development In Starck J. M., & Ricklefs R. E. (Eds.), Avian growth and development: Evolution within the altricial‐precocial spectrum (pp. 31–58). NY: Oxford University Press.

[ece33524-bib-0031] Russell, A. F. (2016). Chestnut‐crowned babblers: Dealing with climatic adversity and uncertainty in the Australian arid zone In Koenig W. D., & Dickinson J. L. (Eds.), Cooperative breeding in vertebrates: Studies of ecology, evolution and behavior (pp. 150–164). Cambridge, MA: Cambridge University Press MA.

[ece33524-bib-0032] Russell, A. F. , Portelli, D. J. , Russell, D. J. F. , & Barclay, H. (2010). Breeding ecology of the chestnut‐crowned babbler: A cooperative breeder in the desert. Emu, 110, 324–331.

[ece33524-bib-0033] Schilzeth, H. (2010). Simple means of increasing interpretability of regression coefficients. Methods in Ecology and Evolution, 1, 103–113.

[ece33524-bib-0034] Serventy, D. L. (1971). Biology of desert birds In Farner D. S., King J. R., Charles K. C., & Parkes K. C. (Eds.), Avian biology, Vol. 1 (pp. 287–339). New York, NY: Academic Press.

[ece33524-bib-0035] Skutch, A. F. (1957). The incubation patterns of birds. Ibis, 99, 430–458.

[ece33524-bib-0036] Skutch, A. F. (1962). The constancy of incubation. The Wilson Bulletin, 71, 115–152.

[ece33524-bib-0037] Tatner, P. , & Bryant, D. M. (1993). Interspecific variation in daily energy expenditure during avian incubation. Journal of Zoology, 231, 215–232.

[ece33524-bib-0038] Thackeray, S. J. , Henrys, P. A. , Hemming, D. , Bell, J. R. , Botham, M. S. , Burthe, S. , … Wanless, S. (2016). Phenological sensitivity to climate across taxa and trophic levels. Nature, 535, 241–245. 27362222 10.1038/nature18608

[ece33524-bib-0039] Tinbergen, J. M. , & Williams, J. B. (2002). Energetics of incubation In Deeming D. C. (Ed.), Avian incubation: Behaviour, environment and evolution (pp. 299–313). UK: Oxford University Press.

[ece33524-bib-0040] Tulp, I. , & Schekkerman, H. (2006). Time allocation between feeding and incubation in uniparental arctic‐breeding shorebirds: Energy reserves provide leeway in a tight schedule. Journal of Avian Biology, 37, 207–218.

[ece33524-bib-0041] Vasseur, D. A. , DeLong, J. P. , Gilbert, B. , Greig, H. S. , Harley, C. D. G. , McCann, K. S. , … O'Connor, M. I. (2014). Increased temperature variation poses a greater risk to species than climate warming. Proceedings of the Royal Society of London Series B: Biological Sciences, 281, 20132612, 1–8. 10.1098/rspb.2013.2612 PMC3924069 24478296

[ece33524-bib-0042] Visser, M. E. , Both, C. , & Lambrechts, M. M. (2004). Global climate change leads to mistimed avian reproduction. Advances in Ecological Research, 35, 89–110.

[ece33524-bib-0043] Visser, M. E. , van Noordwijk, A. J. , Tinbergen, J. M. , & Lessells, C. M. (1998). Warmer springs lead to mismatched reproduction. Proceedings of the Royal Society of London Series B: Biological Sciences, 265(1408), 1867–1870.

[ece33524-bib-0501] Visser, M.E. , te Marvelde. L . & Lof, M.E . (2015). Adaptive phenological mismatches of birds and their food in a warming world. Journal of Ornithology, 153, 75–84.

[ece33524-bib-0044] Vleck, C. M. (1981). Energetic cost of incubation in the zebra finch. The Condor, 83, 229–237.

[ece33524-bib-0045] Webb, D. (1987). Thermal tolerance of avian embryos: A review. The Condor, 89, 874–898.

[ece33524-bib-0046] Williams, J. B. (1996). Energetics of avian incubation In Carey C. (Ed.), Avian energetics and nutritional ecology (pp. 375–416). NY: Chapman & Hall.

[ece33524-bib-0047] Young, C. M. , Browning, L. E. , Savage, J. L. , Griffith, S. C. , & Russell, A. F. (2013). No evidence for deception over allocation to brood care in a cooperative bird. Behavioral Ecology, 24(1), 70–81.

[ece33524-bib-0048] Zuur, A. F. , Ieno, E. N. , Walker, N. , Saveliev, A. A. , & Smith, G. M. (2009). Mixed effects models and extensions in ecology. New York, NY: R. Springer.

